# Prorocentrolide-A from Cultured *Prorocentrum* *lima* Dinoflagellates Collected in Japan Blocks Sub-Types of Nicotinic Acetylcholine Receptors

**DOI:** 10.3390/toxins10030097

**Published:** 2018-02-28

**Authors:** Muriel Amar, Rómulo Aráoz, Bogdan I. Iorga, Takeshi Yasumoto, Denis Servent, Jordi Molgó

**Affiliations:** 1 Commissariat à l’énergie Atomique et aux énergies Alternatives (CEA), Institut des Sciences du Vivant Frédéric Joliot, Service d’Ingénierie Moléculaire des Protéines, Université Paris-Saclay, Bâtiment 152, 91191 Gif-sur-Yvette, France; muriel.amar@cea.fr (M.A.); romulo.araoz@cea.fr (R.A.); denis.servent@cea.fr (D.S.); 2 Institut des Neurosciences Paris-Saclay, UMR 9197 CNRS-Université Paris-Sud, 91198 Gif-sur-Yvette, France; 3 CNRS, Institut de Chimie des Substances Naturelles, UPR 2301, Labex LERMIT, 91198 Gif-sur-Yvette, France; bogdan.iorga@cnrs.fr; 4 Japan Food Research Laboratories, 6-11-10 Nagayama, Tama, Tokyo 206-0025, Japan; yasumotot@jfrl.or.jp

**Keywords:** prorocentrolides, dinoflagellate toxin, cyclic imine toxins, nicotinic acetylcholine receptors, *Xenopus* oocytes, nicotinic currents, binding assays, molecular docking

## Abstract

Prorocentrolides are members of the cyclic imine phycotoxins family. Their chemical structure includes a 26-membered carbo-macrocycle and a 28-membered macrocyclic lactone arranged around a hexahydroisoquinoline that incorporates the characteristic cyclic imine group. Six prorocentrolides are already known. However, their mode of action remains undetermined. The aim of the present work was to explore whether prorocentrolide-A acts on nicotinic acetylcholine receptors (nAChRs), using competition-binding assays and electrophysiological techniques. Prorocentrolide-A displaced [^125^I]α-bungarotoxin binding to *Torpedo* membranes, expressing the muscle-type (α1_2_β1γδ) nAChR, and in HEK-293 cells, expressing the chimeric chick neuronal α7-5HT_3_ nAChR. Functional studies revealed that prorocentrolide-A had no agonist action on nAChRs, but inhibited ACh-induced currents in *Xenopus* oocytes that had incorporated the muscle-type α1_2_β1γδ nAChR to their membranes, or that expressed the human α7 nAChR, as revealed by voltage-clamp recordings. Molecular docking calculations showed the absence of the characteristic hydrogen bond between the iminium group of prorocentrolide-A and the backbone carbonyl group of Trp147 in the receptor, explaining its weaker affinity as compared to all other cyclic imine toxins. In conclusion, this is the first study to show that prorocentrolide-A acts on both muscle and neuronal nAChRs, but with higher affinity on the muscle-type nAChR.

## 1. Introduction

A large number of marine dinoflagellate species of the genus *Prorocentrum*, distributed in benthic and planktonic habitats, have been described [1,2]. Among these dinoflagellates *Prorocentrum lima* and *Dinophysis* spp. are abundantly distributed worldwide in tropical to temperate and cold waters regions [3,4,5], and are known to produce a number of bioactive compounds [6]. These compounds include: okadaic acid and its analogues [7,8], and the associated dinophysistoxins (DTX1 and DTX2) [9,10], which can also occur as complex assortments of esters derivatives [11], and are the main toxins responsible for incidents of diarrheic shellfish poisoning (DSP) [12,13,14,15,16,17]. Okadaic acid and its analogues are highly-specific inhibitors of serine/threonine protein phosphatases PP1 and PP2A [18,19,20]. Additionally, these toxins are potent tumor promoters [21,22,23,24], and can induce genotoxicity in some cell types [25,26,27,28]. 

The dinoflagellate *Prorocentrum lima* has been recognized to produce also some other bioactive compounds including: the prorocentrolide [29], spiro-prorocentrimine [30], prorocentin [31], the formosalides [32], and the limaol polyketide [33]. Interestingly, the analysis of extracts obtained from cultured *Prorocentrum lima* dinoflagellates allowed the first chemical and structural identification of the macrocyclic compound named prorocentrolide, which is a “fast-acting toxin” due to the rapid onset of neurological symptoms, followed by paralysis and death after intraperitoneal administration in mouse bioassays for detecting lipid soluble toxins [29]. Such symptoms were completely different from those reported with diarrheic toxins. Further studies using bioassay-guided fractionation of extracts of the dinoflagellate *Prorocentrum maculosum* Faust, allowed the chemical characterization of prorocentrolide-B in those extracts [34]. Recently, a new tropical toxic benthic dinoflagellate species (*Prorocentrum caipirignum*), related to the *P. lima* species complex, has been reported to produce both okadaic acid and the fast acting prorocentrolide toxin [35]. 

Prorocentrolides are members of the cyclic imine family of phycotoxins that are known to contaminate seafood, and which includes the gymnodimines, spirolides, pinnatoxins, portimine, pteriatoxins, and spiro-prorocentrimine (reviewed in [36,37,38,39]). Some of these cyclic imine toxins have been reported to be potent antagonists of muscle- and neuronal-types of nicotinic acetylcholine receptors (nAChRs), as recently reviewed [40]. 

**Figure 1:**
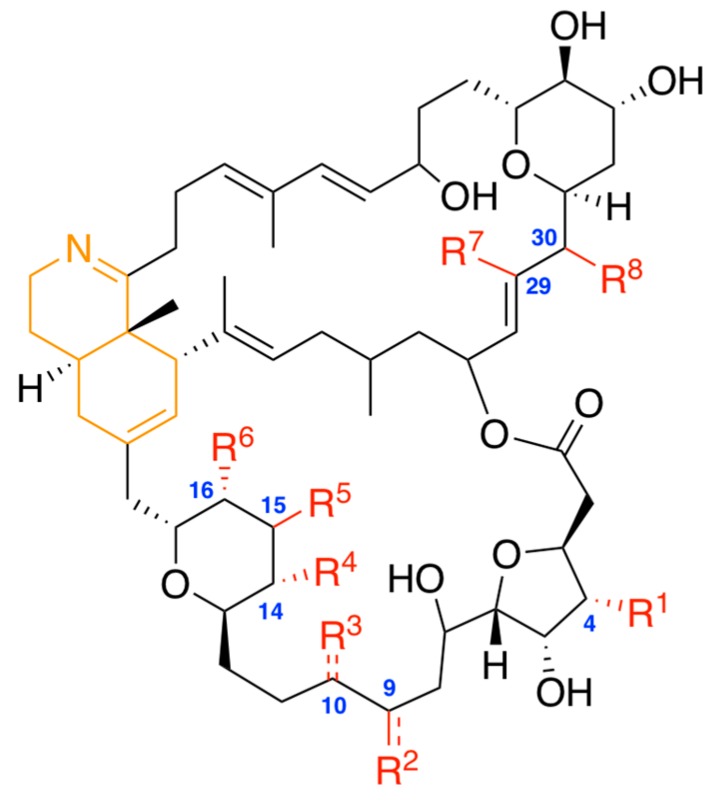
General chemical structure of prorocentrolides and analogues. The nature of substituents R^1^ to R^8^ (colored in red) is detailed in Table 1. The cyclic imine group is colored in orange.

The chemical structure of prorocentrolides comprises a 26-membered carbo-macrocycle and a 28-membered macrocyclic lactone arranged around a hexahydroisoquinoline that incorporates the characteristic cyclic imine group (Figure 1). At present, as shown in Table 1, six prorocentrolides are already known which differ by their chemical structures. 

Despite the fact that prorocentrolide-A was the first cyclic imine toxin discovered (already in 1988 [29]), and that it has been reported in several *Prorocentrum* species [34,35], its mode of action remains unknown. There are several reasons for this: (i) the production by dinoflagellates is very limited; (ii) not all *P. lima* species seem to produce the compound; and (iii) the ecological conditions that may favor the production of prorocentrolides remain unknown. 

To the best of our knowledge the possibility that prorocentrolide-A could target nAChRs has not been previously investigated. Consequently, the aim of the present study was to examine whether prorocentrolide-A acted on nAChRs. For this, we used heterologous expression of nAChRs on both *Xenopus laevis* oocytes and HEK-293 cells, and *Torpedo* electric organ membranes together with voltage-clamp recordings and binding assays. The present study shows for the first time that prorocentrolide-A acts on both muscle-type (α1_2_β1γδ) and neuronal human α7 and chick chimeric α7-5HT_3_ nAChRs. Differences in affinity are discussed based on molecular docking calculations performed with the extracellular domain of these receptors subtypes.

## 2. Results

### 2.1. Effect of Prorocentrolide-A on Xenopus Oocytes after Heterologous Expression of the Human α7 nAChR

**Figure 2:**
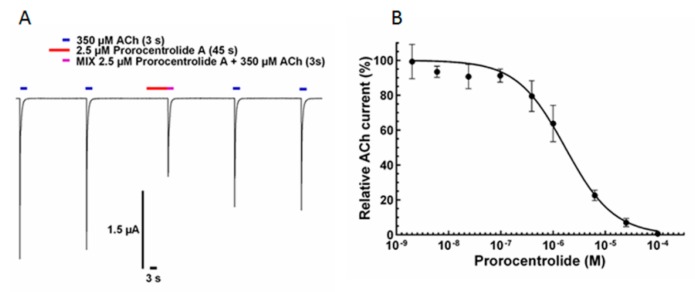
Effect of prorocentrolide-A on the human α7 nAChR expressed in *Xenopus* oocytes. (**A**) Typical inward nicotinic currents evoked by ACh (350 μM) applied for 3 s and recorded at −60 mV holding membrane potential. The blue tracings above the current traces denote the perfusion of ACh. The red tracing (above the current trace) denotes the perfusion of 2.5 μM prorocentrolide-A. Note that no current was evoked by the perfusion of the prorocentrolide alone, which indicates that it has no direct agonist action on the α7-receptor, while when applied together with ACh (red and blue tracing, MIX, 3rd current trace from left) a block of the peak inward current was observed. The washout of the prorocentrolide from the medium by the fast perfusion system allowed a partial recovery of the ACh evoked current (4th and 5th current tracings) as compared to the control currents (first two tracings). (**B**) Concentration-dependent inhibition of ACh-elicited nicotinic currents by prorocentrolide-A in oocytes expressing the human α7 nAChR. Peak amplitudes of ACh-evoked currents (mean ± SEM), recorded at −60 mV in the presence of the prorocentrolide were normalized to control currents, and fitted to the Hill equation (*n*H = 0.93). The concentration of ACh used was the EC_50_ determined.

The effect of prorocentrolide-A was investigated in *Xenopus* oocytes that have been previously transfected with the human α7 nAChR. Two to five days after transfection, oocytes were impaled with two microelectrodes and voltage-clamped at –60 mV holding membrane potential. Perfusion of 350 μM acetylcholine (ACh) (which corresponds to the EC_50_ for ACh) for 3 s elicited phasic inward nicotinic currents, which varied in peak amplitude between 0.2 μA and 3 μA among oocytes studied, and rapidly inactivated (*n* = 45 oocytes tested from eight different *Xenopus* donors). As shown in Figure 2A, twin control perfusions of ACh evoked the typical phasic inward currents that had similar amplitudes when spaced by a 3 min time interval. Prorocentrolide-A when perfused at concentration ranging from 3 × 10^−9^ M to 4 × 10^−4^ M had no detectable agonist action on the α7 nAChR (Figure 2A), but dose-dependently decreased the peak amplitude of the ACh-elicited current, as shown in Figure 2B. The inhibitory action of Prorocentrolide-A had an IC_50_ = 1660 nM (1641–1680 nM, 95% confidence intervals, 45 oocytes, from eight *Xenopus* donors).

### 2.2. Effect of Prorocentrolide-A on *Xenopus* Oocytes after Microtransplantation of *Torpedo* Muscle-Type α1_2_β1γδ nAChR

Further studies were performed on *Xenopus* oocytes that have been micro-transplanted with purified membranes from the electric organ of *Torpedo* containing the muscle-type (α1_2_β1γδ) nAChR. After a few days, once the α1_2_β1γδ nAChR have been incorporated to the oocyte membrane, oocytes were voltage-clamped at −60 mV. For nAChR activation, the perfusion of ACh was used at the concentration corresponding to its experimentally-determined EC_50_ (25 μM). Prorocentrolide-A, by itself, did not evoke any inward current in the range of concentrations tested (10^−10^–10^−4^ M) indicating that it had no agonist action on the α1_2_β1γδ nAChR. However, it dose-dependently reduced the peak amplitude of the ACh-evoked currents, with an IC_50_ = 185.7 nM (165–209 nM, 95% confidence intervals, 36 oocytes, from nine *Xenopus* donors).

### 2.3. Competition-Binding Assays between Prorocentrolide-A and Radiolabeled α-Bungarotoxin

**Figure 3:**
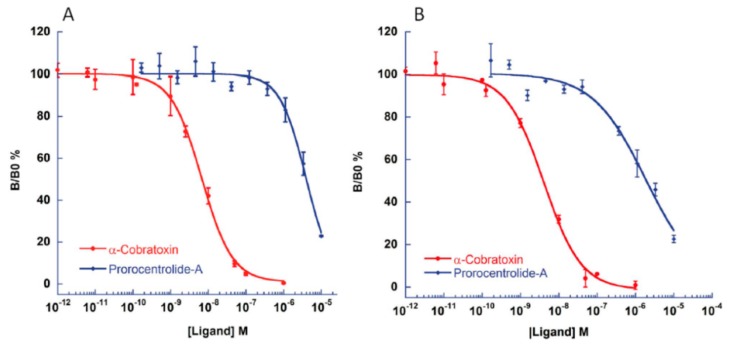
Prorocentrolide-A and α-cobratoxin displaced, in a concentration-dependent manner, the specific [^125^I]α–BTX binding to HEK-293 cells, expressing the chimeric chick neuronal α7-5HT_3_ nAChR (**A**) and to *Torpedo* membranes expressing the muscle-type α1_2_β1γδ nAChR; and (**B**) each point in the curves represents the mean ± SEM of three different experiments performed in duplicate.

Additional information on the interaction between prorocentrolide-A and nAChRs was obtained by competition-binding assays at equilibrium, which allowed the characterization of the binding affinity and the antagonist potency of the phycotoxin. For this, purified *Torpedo* membranes expressing the muscle-type α1_2_β1γδ nAChR, and HEK-293 cells expressing the chimeric chick neuronal α7-5HT_3_ nAChR were used together with [^125^I]α-BTX, as radiotracer. Figure 3, shows that prorocentrolide-A concentration-dependently displaced [^125^I]α-BTX from the *Torpedo* muscle-type receptor, and from neuronal α7-5HT_3_ nAChR expressed in HEK-293 cells, but with much less efficacy than the high affinity α-cobratoxin from *Naja* *kaouthia*.

From the binding-competition curves of Figure 3, it was possible to calculate the affinity constants (see the Materials and Methods section) shown in Table 2. Prorocentrolide-A interacted with nanomolar affinity with the muscle-type α1_2_β1γδ nAChR, but with much lower affinity than the α-cobratoxin which exhibited sub-nanomolar affinity on this receptor subtype. As disclosed on Table 2, prorocentrolide-A interacted with much lower affinity with the neuronal α7-5HT_3_ nAChR.

### 2.4. Molecular Docking Interactions between Prorocentrolide-A and the Extracellular Domain of Muscle-Type α1_2_β1γδ and Neuronal α7 nAChR

**Figure 4:**
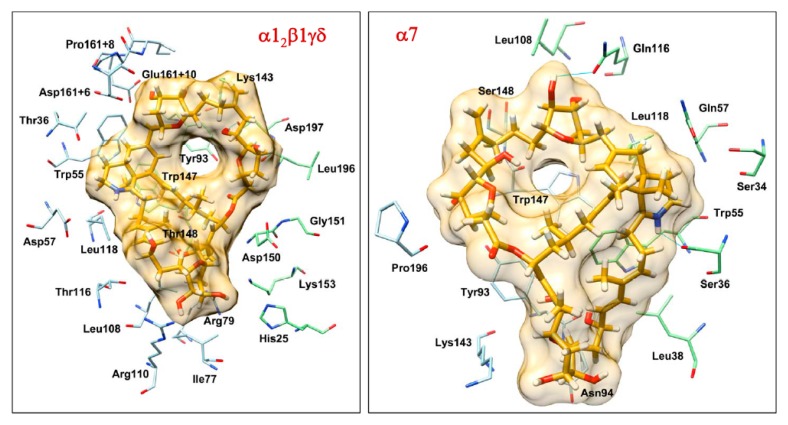
Docking conformations of prorocentrolide-A at the subunit interface of muscle-type (**left**) and neuronal α7 nAChR (**right**). Residues within 4 Å from the ligand are shown. Loop C is hidden for more clarity.

The protocol described previously [41,42,45] was used for the docking of prorocentrolide at the intersubunit interface of the extracellular domain of α1_2_β1γδ and α7 nAChRs, with one significant change. The existing conformations of the nAChR could not accommodate the unusual size of the prorocentrolide-A ligand. Therefore, the docking calculations were carried out in the absence of the C loop, which was added subsequently in a conformation that was compatible with the presence of the ligand within the binding site. The best docking conformations are presented in Figure 4.

## 3. Discussion

Using both electrophysiological voltage-clamp techniques and competition ligand-binding assays, present results show that prorocentrolide-A targets both heteropentameric muscle-type α1_2_β1γδ nAChR and homopentameric neuronal-α7 nAChRs. These ligand-gated ion channels mediate fast transmission at the skeletal neuromuscular junction and in the central and peripheral nervous systems, respectively (reviewed in [46,47]). 

Prorocentrolide-A, in contrast to ACh (the endogenous agonist of nAChRs), had no agonist properties on both α1_2_β1γδ and α7 nAChRs incorporated or expressed in the oocyte membrane, respectively, but blocked the inward current evoked by ACh. Ligand-binding assays, performed on membranes and cells expressing the different nAChR subtypes, using [^125^I]α-BTX and standard methods, allowed a better understanding of the interaction between prorocentrolide-A and nAChRs. These competition-binding assays demonstrated the concentration-dependent displacement of [^125^I]α-BTX from *Torpedo* membranes expressing the muscle-type α1_2_β1γδ nAChR and from HEK-293 cells expressing the chicken chimeric α7-5HT_3_ neuronal nAChR. These results indicate that prorocentrolide-A is a direct competitive antagonist in both nAChR subtypes, although much less active than previously-examined cyclic imine toxins and the α-cobratoxin herein studied. As shown in Table 2, prorocentrolide-A exhibited the lowest affinity on the α7-5HT_3_ neuronal nAChR when compared to the muscle-type α1_2_β1γδ nAChR. 

The higher affinity of prorocentrolide-A to muscle-type nAChR is an important factor contributing to the acute toxicity of this compound. The acute toxic symptoms observed in mice following prorocentrolide-A administration [29] included a rapid onset for skeletal muscle paralysis from the hind legs and respiratory muscles which led to death due to respiratory arrest. Such actions are likely due to the block of nAChRs in the endplate region of skeletal muscles. There was a critical dose-dependency, below which surviving mice recovered completely. The lack of description of initial hyperactivity periods, following the administration of prorocentrolide-A, probably reflects the poor affinity of the compound to the α7 nAChRs, here reported. The initial hyperactivity periods characterizing the action of most cyclic imine toxins are likely due to a direct effect on the central nervous system of rodents [48]. 

Important developments have been made in the structural and molecular characterization of nAChRs and in the understanding of the molecular pharmacological profile of cyclic imine toxins (reviewed in [40]). A major step in our knowledge of the structural determinants came from the X-ray crystal structure of some cyclic imine toxins in complex with the acetylcholine binding protein (AChBP) [43,49]. AChBPs are water-soluble pentameric proteins, representing structural and functional homologues of the amino-terminal extracellular ligand-binding domain of nAChRs [49,50]. Well-preserved amino acid residues occurring in the nAChR family are existent in the AChBPs, comprising those that are significant for the ligand binding to agonists such as ACh, carbamylcholine, nicotine, as well as for competitive antagonists, for instance, d-tubocurarine and α-BTX [50,51,52,53,54,55,56] and, consequently, have similar pharmacological properties.

The complexes of prorocentrolide-A with α1_2_β1γδ and α7 nAChRs obtained by docking (Figure 4) show two different binding modes. Prorocentrolide-A interacts mostly through the convex side with the α1_2_β1γδ nAChR, thus establishing an important number of stabilizing hydrophobic and polar interactions with the neighboring residues (Figure 4, left). The hydrogen bond between the hydroxyl group in position 7 with the side chain of Asp197 also seems to be a key stabilizing interaction. Overall, the numerous favorable interactions and the good shape complementarity between the receptor and the ligand can explain the relative good affinity of prorocentrolide-A for the α1_2_β1γδ nAChR. On the other hand, prorocentrolide-A is oriented with its concave side towards the binding site of α7 nAChR, which limits the number of possible interactions (Figure 4, right). These interactions are observed mainly at the extremities of the ligands (e.g., hydrogen bonds between iminium group and Ser36 and between the hydroxyl in position 14 and Gln116). Therefore, the limited number of interactions and the absence of shape complementarity are responsible for the lower affinity of prorocentrolide-A for the α7 nAChR. It is worth noting that the hydrogen bond interaction between the iminium group and the backbone of Trp147, a general feature of complexes between spiroimine toxins and nAChRs, is absent from the complexes of prorocentrolide-A with the nAChRs studied. These results also evidenced the originality of prorocentrolide-A in terms of chemical structure and nAChR binding mode, which is completely different from those of agonists (e.g., acetylcholine), or other known antagonists (e.g., spiroimine toxins, α-cobratoxin) of nAChRs.

## 4. Conclusions

The present study is the first to show that prorocentrolide-A blocks the *Torpedo* muscle-type α1_2_β1γδ nAChR and the human α7 nAChR incorporated or expressed, respectively, in *Xenopus* oocytes. In addition, competitive-binding studies demonstrated that the toxin displaced [^125^I]α-BTX from α1_2_β1γδ nAChRs from *Torpedo* electric organ membranes and chimeric α7-5HT_3_ expressed in HEK-293 cells. Prorocentrolide-A exhibited sub-micromolar affinity for the muscle-type nAChR and was more potent than for the neuronal α7-5HT_3_ nAChR. Compared to other cyclic imine toxins, prorocentrolide-A is the less active, and molecular docking calculations showed that this is due, at least in part, to the absence of the characteristic hydrogen bond between the iminium group of prorocentrolide-A and the backbone carbonyl group of Trp147 in the receptor that is present in all other cyclic imine toxins.

## 5. Materials and Methods

### 5.1. Materials and Reagents

[^125^I]α-Bungarotoxin ([^125^I]α-BTX) (210–250 Ci·mmol^−1^) and the scintillation solution (Ultima Gold F) were purchased from PerkinElmer (Courtaboeuf, France). Ethyl-3-amino benzoate methanesulfonate, ethylene diamine tetraacetic acid (EDTA), acetylcholine chloride, tricaine, and other chemicals were from Sigma-Aldrich (Saint Quentin Fallavier, France), or other standard sources. The α-cobratoxin from *Naja kaouthia* was obtained by recombinant expression, refolded, and purified in our laboratory, as described previously (see [57]).

### 5.2. Animals and Biological Materials

Adult female *Xenopus laevis* frogs were purchased at the Centre de Ressources Biologiques Xenopes—CNRS (Université de Rennes 1, Rennes, France), and *Torpedo marmorata* fish at the Service Modeles Biologiques of the Station Biologique de Roscoff (Roscoff, France). All animal studies were performed in accordance with the guidelines established by the French Council on animal care “Guide for the Care and Use of Laboratory Animals”: EEC86/609 Council Directive—Decree 2001-131. The protocols were approved by the French Departmental Direction of Animal Protection (No. A91-453 to Rómulo Aráoz) and the CNRS animal care and use committee. All experiments were approved by the Animal Care and Use Committee of the French Ministry of National Education, High Education and Research (identification code: APAFIS#5310-2016042016067330 v3; date of approval: 10 November 2016).

**Figure 5:**
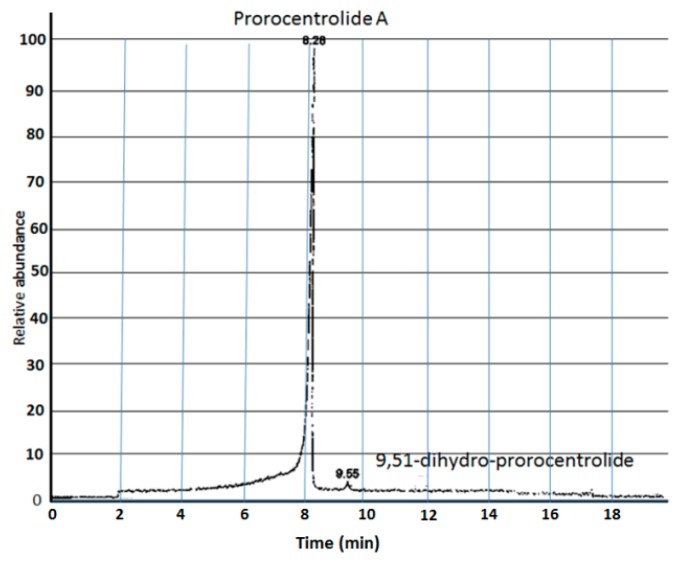
LC-MS chromatogram of the sample used in the experiments here reported. Note the relative abundance of prorocentrolide-A. The small peak component revealed at 9.55 min corresponds to the 9,51-dihydroprorocentrolide.

The cDNAs coding for chick chimeric α7-5HT_3_ and human α7 nAChR were kindly provided by Dr. Pierre-Jean Corringer (Pasteur Institute, Paris, France), and by Professor Isabel Bermudez (Oxford Brookes University, Oxford, UK). Prorocentrolide-A was obtained from extracts of cultured *Prorocentrum lima* dinoflagellates, isolated at Sesoko Island, Okinawa, Japan, prepared as previously reported [29], and provided by Professor Takeshi Yasumoto. Figure 5, shows a liquid chromatography-mass spectrometry (LC-MS) chromatogram of the sample used in the present experiments. The purity of the sample was further checked by thin layer chromatography which showed only a single spot (not shown).

### 5.3. Expression of the Human α7 nAChR in *Xenopus* Oocytes

Oocytes were removed from mature female *Xenopus laevis* frogs under anesthesia, as previously described [41], and placed in a medium devoid of calcium and containing (in mM): NaCl, 88; KCl, 2.5; MgCl_2_, 1; and HEPES, 5 (pH 7.6). After substantial washing with this solution, oocytes were transferred to a Barth’s solution containing (mM): NaCl, 88; KCl, 1; MgSO_4_, 0.33; CaCl_2_, 0.41; MgSO_4_, 0.82; Ca(NO_3_)_2_, 0.33; NaHCO_3_, 2.4; and HEPES, 10 (pH = 7.2) supplemented with 0.1 μg·mL^−1^ kanamycine. Selected oocytes (stage V–VI) were manually defolliculated, and microinjected with the aid of a Nanoliter 2000 Micro4 Controller (World Precision Instruments, Inc., Hertfordshire, UK) with 50 nL of human α7 mRNA (0.1 μg·μL^−1^). Microinjected oocytes were incubated at 18 °C in Barth’s solution, and voltage-clamp recordings were performed 3–4 days later. 

### 5.4. Microtransplantation of *Torpedo* Muscle-Type nAChR to *Xenopus* Oocytes

Surgical removal of the electric organs of *Torpedo marmorata* fish was performed under 0.03% tricaine anaesthesia diluted in seawater. *Torpedo* electric organs were sliced and purified membranes enriched in the α1_2_β1γδ nAChR were made at 4 °C in 5 mM glycine, using methods described previously [43,58]. Aliquots of the purified membranes were stored at −80 °C until use. Microtransplantation of *Torpedo* nAChR [58,59] consisted in a single microinjection of a membrane suspension (50 nL at 3.5 mg·mL^−1^ protein) into the oocyte cytoplasm using a Nanoliter 2000 Micro4 Controller mounted on a microscope (World Precision Instruments, Inc., Hertfordshire, UK), as previously described [43].

### 5.5. Voltage-Clamp Recordings in *Xenopus* Oocytes

A two-microelectrode voltage-clamp amplifier (OC-725B, Warner Instrument Corp., Hamden, CT, USA) was used to record currents flowing through nAChRs upon activation or inhibition. The voltage and current microelectrodes were filled with a solution of 3 M KCl and had tip resistances comprised between 0.5 and 1.5 MΩ. Oocytes were voltage-clamped at –60 mV holding membrane potential. A pCLAMP-9/Digidata-1322A system (Molecular Devices, Union City, CA, USA) was used for data acquisition and recording. The recording chamber had a capacity of 300 μL, and was superfused at a rate of 8 mL·min^−1^ at 20 °C with a modified Ringer’s solution containing (mM): NaCl, 100; KCl, 2.8; MgCl_2_, 1; BaCl_2_, 0.3; and HEPES, 5 (pH 7.4), where BaCl_2_ replacement to CaCl_2_ prevents secondary activation of Ca^2+^-dependent chloride current [41,60]. A multi-valve perfusion system (VC-6, Warner Instruments Corp., Hamden, CT, USA) controlled by a PC computer was used for exchanging solutions, and to perfuse ACh or prorocentrolide-A. ACh was perfused during 3 s periods in oocytes expressing the human α7 nAChR, and for 15 s periods in oocytes having incorporated the *Torpedo* α1_2_β1γδ nAChR into their membrane. A 3 min interval was used between consecutive ACh applications, to ensure nAChR recovery from desensitization. Dose-response inhibition curves were constructed, as detailed previously [42] using GraphPad Prism 6.05 (GraphPad Software, Inc., San Diego, CA, USA, 2013) software.

### 5.6. Expression of nAChRs in Human Embryonic Kidney Cells and Binding Assays

The chimeric chick cDNA of the α7-5HT3 nAChR was transfected into human embryonic kidney (HEK-293) cells by methods previously described [61,62]. Briefly, the cDNA (15 μg of α7-5HT_3_) was transfected by calcium precipitation with a meticulous pH control (6.95). Cells were placed at 37 °C under 5% CO_2_, and 48 h after transfection were collected in a phosphate-buffered saline (PBS) with 5 mM EDTA, and suspended in 3 mL/plate of this buffer for binding experiments. Cell density was adjusted to bind specifically about 10% of the radioligand.

Binding assays with *Torpedo* or α7-5HT_3_ nAChRs were performed at equilibrium on 96-well plates. Membranes were incubated for 4 h with distinct concentrations of prorocentrolide-A or α-cobratoxin and [^125^I]α-BTX (0.5–1 nM). Nonspecific binding was performed in the presence of 1 μM α–cobratoxin. All the reactions were stopped by filtration of the 96-well simultaneously through a GF/C plate pre-soaked in 0.5% polyethylenimine, using a FilterMate harvester (PerkinElmer, France). The filters were washed twice with ice-cold buffer (PBS), dried and the bound radioactivity was counted, after the addition of 25 μL of MicroScint 0 per well, by scintillation spectrometry on a TopCount beta counter (PerkinElmer, France). IC_50_ values were determined by fitting the competition data by the empirical Hill’s equation and converting to Ki constants using the equation: Ki = IC_50_/(1 + L*/Kd) [63]. The Kd for α-BTX was 50 pM and 5 nM on muscle-type and α7 receptors, respectively. All experiments were performed at least three times in duplicate.

### 5.7. Molecular Modeling

Homology models of the extracellular domain of human α7 and *Torpedo* α1_2_β1γδ nAChRs subtypes were constructed using Modeller [64], and the *Aplysia californica* acetylcholine binding protein (AChBP) crystal structure as a template (Protein Data Bank code 2WZY) [43]. Three-dimensional structures of the ligand were generated using Corina 3.6 (Molecular Networks GmbH, Erlangen, Germany, 2016). The docking procedure was carried out in two steps: (i) conformational search of all possible prorocentrolide stereoisomers, using MacroModel (Schrödinger LLC, Portland, OR, USA) and (ii) molecular docking using Gold (Cambridge Crystallographic Data Centre, Cambridge, UK) and the GoldScore scoring function of the resulting conformers at the subunit interfaces of α7 and α1_2_β1γδ homology models. The binding site, defined as a 20 Å radius sphere, was centered on the backbone oxygen atom of Trp147. All other parameters had default values. Initial docking calculations provided no useful results due to the unusual size of the ligand and binding site hindering by the C loop. A second round of docking was carried out with the C loop (residues RFYECCKEPY and VYYTCCPDTPY, respectively) removed. The C loop was reconstructed afterwards using Modeller [63] in the presence of the ligand in the binding site. The receptor-ligand complex images were produced using UCSF Chimera [65].

## Figures and Tables

**Table 1:** Prorocentrolides and analogues that have been reported to date.

Prorocentrolide	R^1^ *	R^2^	R^3^	R^4^	R^5^	R^6^	R^7^	R^8^
Prorocentrolide-A	H	CH_2_=	H	OH	OH	H	CH_3_	OH
30-sulfate prorocentrolide	H	CH_2_=	H	OH	OH	H	CH_3_	OSO_3_H
4-hydroxy prorocentrolide	OH	CH_2_=	H	OH	OH	H	CH_3_	OH
9,51-dihydro prorocentrolide	H	CH_3_	H	OH	OH	H	CH_3_	OH
14-*O*-acetyl-4-hydroxy prorocentrolide	OH	CH_2_=	H	OC(=O)CH_3_	OH	H	CH_3_	OH
Prorocentrolide-B	OSO_3_H	CH_3_	CH_2_=	OH	H	OH	H	OH

* See Figure 1 for the general chemical structure.

**Table 2:** Affinity constants (Ki ± SEM, nM) of prorocentrolide-A, and the comparison to the nicotinic antagonist α-cobratoxin from *Naja kaouthia,* and to other cyclic imine toxins previously studied. Data was obtained in competition binding assays at equilibrium on *Torpedo* muscle-type α1_2_β1γδ and chimeric chick α7-5HT_3_ nAChR. Values on Hill coefficients (*n*H) are included below Ki values.

Nicotinic Antagonist	α1_2_β1γδ	α7-5HT_3_	Reference
Prorocentrolide-A	81.70 ± 16.1 ^a^	3380.0 ± 695	This work
	(*n*H = 0.66 ± 0.27 ^b^)	(*n*H = 1.19 ± 0.4)	
α-cobratoxin	0.397 ± 0.153	5.60 ± 0.19	This work
	(*n*H = 0.89 ± 0.06)	(*n*H = 1.02 ± 0.07)	
13,19-didesmethyl spirolide C	0.017 ± 0.003	0.22 ± 0.06	[41]
20-methyl spirolide G	0.028 ± 0.005	0.11 ± 0.08	[42]
13-desmethyl spirolide C	0.080 ± 0.002	0.53 ± 0.08	[43]
Gymnodimine-A	0.23 ± 0.08	0.33 ± 0.08	[44]
Pinnatoxin-A	2.80 ± 0.03	0.35 ± 0.04	[45]

^a^ Data are presented as the mean ± SEM from three distinct experiments performed in duplicate; ^b^ Data on the Hill coefficients (*n*H) are presented as the mean ± SEM from three distinct experiments performed in duplicate.
